# How Workplace Bullying Jeopardizes Employees’ Life Satisfaction: The Roles of Job Anxiety and Insomnia

**DOI:** 10.3389/fpsyg.2019.02292

**Published:** 2019-10-24

**Authors:** Shazia Nauman, Sania Zahra Malik, Faryal Jalil

**Affiliations:** ^1^Riphah School of Business and Management, Riphah International University, Lahore, Pakistan; ^2^Institute of Business Administration (IBA), University of the Punjab, Lahore, Pakistan; ^3^Institute of Quality and Technology Management (IQTM), University of the Punjab, Lahore, Pakistan

**Keywords:** workplace bullying, job-related anxiety, insomnia, life satisfaction, conservation of resources theory

## Abstract

Drawing on conservation of resources (COR) theory, this study examined the underlying mechanism through which workplace bullying (WB) affects employees’ life satisfaction *via* job-related anxiety and insomnia. Time-lagged data were collected at two points in time from 211 doctor interns working in various hospitals in Pakistan. Our results fully supported a proposed serial multiple-mediator model. Workplace bullying was indirectly related to life satisfaction *via* job-related anxiety and insomnia. This study provides evidence of a spillover effect as to how workplace bullying increases employees’ job-related anxiety which in turn leads to insomnia resulting in reduced employees’ life satisfaction. The present study extends research on workplace bullying to display its theoretical as well as empirical effects on life satisfaction. It demonstrates that workplace bullying as an occupational and psychological stressor has multiple effects on employees’ life satisfaction through a serial mediation model in the context of a developing country. It further explains that workplace bullying not only affects an employee’s workplace behaviors but also extends to the employee’s overall life satisfaction.

Workplace bullying (WB) has been considered by prior researchers as one of the primary issues for workers’ safety and health and as a key predictor of deteriorating health and well-being among employees because of its severe consequences (Vie et al., 2011; Nielsen and Einarsen, 2012; Einarsen and Nielsen, 2015; Verkuil et al., 2015). Bullying is frequently linked with psychological distress and psychosomatic symptoms (Einarsen et al., 1998; Savicki et al., 2003). Workplace bullying is thus an occupational stressor (Agervold, 2007), causing harmful effects on its sufferers (Einarsen et al., 2003) such as increased job stress (Jenkins et al., 2011), mental disorder (Finne et al., 2011), depression and emotional exhaustion (Varhama and Björkqvist, 2004; Hershcovis and Barling, 2010), aggression (Vega and Comer, 2005), reduced organizational citizenship behaviors and increased organizational retaliatory behaviors (Naseer et al., 2018), work-family conflict (Raja et al., 2017), and workplace deviant behaviors (Litzky et al., 2006). However, little attention has been paid to how WB as a social stressor spills over from workplace to employees’ overall life through a series of psychological consequences. Under the framework of COR theory, this study proposes that workplace bullying triggers a chain of resultant behaviors which cross the boundaries of the workplace to the overall life of a person. Specifically, the study suggests that WB leads to job-related anxiety, which leads to depletion of an important physiological resource, i.e., sleep, thereby causing insomnia, which in turn reduces overall life satisfaction. To the best of our understanding, no prior research has predicted the serial mediation of job-related anxiety and insomnia between WB and life satisfaction.

Bullying refers to offensive, threatening, or insulting behavior that makes others feel disgraced, eventually lowering self-confidence and causing stress (Embree and White, 2010). Bullying behaviors can have a job-related character and involve enforcing inflexible cutoff dates and allocating the unmanageable amount of work, which leads to enhanced feelings of job-related anxiety (Zapf and Einarsen, 2003). Job-related anxiety demonstrates that employees feel tense due to their job and fear about their capability to meet employer’s requirements (Mohr and Puck, 2007; McCarthy et al., 2016). As job-related anxiety is a prominent work-related outcome (Parker and DeCotiis, 1983) and has repercussions for employees, organizations, and society (Barling et al., 2005); it is, therefore, important to inspect the degree to which bullying affects the employees’ overall life *via* job-related anxiety.

Further, research shows that stress and anxiety are blamed for sleep problems as they affect physiological arousal, which hinders the physiological processes embroiled in falling asleep (LeDuc et al., 2000; Vahtera et al., 2007; LeBlanc et al., 2009). Though researchers have determined various harmful consequences of sleep deprivation, we in this study investigate its implications for employees’ life satisfaction. Workplace bullying has been linked to sleep disturbances (Greenberg, 2006; Niedhammer et al., 2009). Different kinds of work demands cut down sleep quality and result in interruptions in sleeping (Kalimo et al., 2000; Åkerstedt et al., 2002a,b). Prior research has revealed that insomnia is linked with negative consequences such as absenteeism and poor work performance (Barnes, 2012). Organizations potentially incur significant cost due to employees’ insomnia problems (Barnes and Van Dyne, 2009). For instance, insomnia is related to employees’ burnout (Vela-Bueno et al., 2008). Scott and Judge (2006) found that insomnia increased feelings of hostility and fatigue and decreased attentiveness and job satisfaction. We posit that the effects of feeling of job-related anxiety have not been examined thoroughly as to how they spill over from work context to employees’ sleep and later on to their life satisfaction. We anticipate a positive relationship of WB with job-related anxiety and insomnia and negative relationship with employees’ life satisfaction *via* job-related anxiety followed by insomnia.

There are several reasons why it is imperative to study life satisfaction as an outcome variable. First, life satisfaction is a part of subjective well-being (Diener et al., 1999; Schimmack et al., 2002) and has been used as a global measure of quality of life. Second, people believe happiness and life satisfaction are more important than money (Diener and Oishi, 2000). Third, a major benefit of life satisfaction is that people have a good feeling about themselves and their lives, which has an impact on their overall well-being. Moreover, it has been associated with psychological and somatic health and has a strong relationship with happiness and job satisfaction (Tait et al., 1989; Judge and Hulin, 1993).

We employed conservation of resources theory (COR) (Hobfoll, 1989) to provide the theoretical basis for this study. COR theory states that “ongoing work demands consistently deplete resources at a faster rate than resources can be replenished” (Freedy and Hobfoll, 1994). Workplace bullying is a stressor, and, employing COR theory, we posit that experiences of WB deplete psychological resources and not only affect an employee’s work-related psychological states, but also spill over to overall life satisfaction. According to COR theory, when employees feel job-related anxiety as a result of workplace bullying, it depletes their energy resources as they are dissatisfied about their job (Hobfoll, 2001). We predict that this feeling of job-related anxiety is negatively related to life satisfaction. Further, we contend that WB is a powerful stressor causing resource loss at multiple levels. When WB causes job-related anxiety, it results in more resource loss leading to poor mental health and insomnia (Rosekind and Gregory, 2010), which eventually affects their life satisfaction.

In sum, this study makes three key contributions. First, our study provides insight into the mechanisms through which workplace bullying as an occupational and psychological stressor leads to job-related anxiety which in turn spills over to employees’ sleep and finally transmits its effect negatively to the employees’ life satisfaction. By addressing workplace bullying-employees’ life satisfaction relationship, we provide organizations an insight into how and when WB transcends employee work and affects the quality of an employee’s overall life satisfaction. Second, we respond to the call of research by Diener et al. (2017) on life satisfaction, who urged to focus on an overall model of well-being and satisfaction, as well-being at work cannot be separated from other aspects of life and vice versa. Third, we extend the research on bullying to exhibit its multiple effects on employees’ life satisfaction through a serially mediated model in the context of a developing country.

## Theory and Hypotheses

### Bullying and Similar Workplace Behaviors

Bullying is a multidimensional and multifaceted phenomenon, which is employed synonymously with similar oppressive workplace behaviors, including workplace harassment (Bowling and Beehr, 2006), emotional abuse (Keashly, 1997), workplace incivility (Estes and Wang, 2008), and abusive supervision (Ng et al., 2012). However, workplace bullying is a distinctive concept which “has been defined as all those repeated actions and practices that are directed to one or more workers, which are unwanted by the victim, done deliberately or unconsciously, but clearly causing humiliation, offense and distress, and that may interfere with job performance and/or cause an unpleasant working environment” (Einarsen, 1999, p. 17). Bullying can be caused by any member of the organization, including supervisors, colleagues, and even by subordinates (Vega and Comer, 2005). It may be faced by both men and women equally, as opposed to sexual harassment, which is usually directed toward women (Einarsen and Nielsen, 2015). There are three major distinct characteristics of workplace bullying that separate it from other similar concepts: which are “(1) the source of mistreatment, (2) the persistence and frequency of treatment, and (3) the superiority (power) of the actor” (Raja et al., 2017, p. 3). The source as already stated can be any member of the organization, at any level; the frequency and persistency are constant and repeated and the bullying person has an impression of superiority over the victim (Hershcovis, 2011). One striking characteristic of workplace bullying is that it is not a stand-alone event; rather it is a series of the ongoing and steadily escalating thread of episodes that creates a cumulative effect on the victim. In isolation, the episodic occurrences may be unfelt and ignored; however, the serial characteristic of WB has a compounding effect (Nielsen and Einarsen, 2012). Therefore, WB is more intense than workplace incivility or social undermining (Hodgins et al., 2014).

Literature shows that workplace bullying is a common workplace phenomenon. A number of studies have proved that workplace bullying is pervasive and exists at all levels, and in all types of professions (Leymann, 1990; Einarsen and Skogstad, 1996; Rayner, 1997; Nielsen et al., 2014). There is sufficient evidence that workplace bullying is a significant stressor that causes increased risk of negative outcomes, particularly health-related problems, such as psychological stress (Nielsen et al., 2012; Magee et al., 2015), sleep deprivation (Hansen et al., 2016), depression and cardiovascular disease (Kivimäki et al., 2003), and reduced well-being (Dehue et al., 2012). It is in fact considered as one of the most “crippling and devastating” workplace problems than any other stressor (Raja et al., 2017, p. 2). It has also been found that it reduces positive individual as well as work-related outcomes. When bullying comes from supervisors, it leads to reduced organizational commitment and organizational citizenship behaviors (Aryee et al., 2007) as well as reduced creativity (Han et al., 2017). It also contributes toward intention to quit as well as actual incidence of quitting the job (Berthelsen et al., 2011), and also increases absenteeism (Dehue et al., 2012). A close correlate, i.e., workplace incivility, reduces performance and it was found that it causes more detrimental outcomes for those who are more committed to the organization, thereby affecting those who are more valuable for the organization (Kabat-Farr et al., 2018).

### Bullying and Life Satisfaction

Literature shows that there are three major indicators of an individual’s subjective well-being, i.e., positive affect, negative affect, and life satisfaction (Linley et al., 2009; Busseri and Sadava, 2011). Out of these three, life satisfaction is the phenomenon of interest in this study, and has been defined as a cognitive assessment of satisfaction with circumstances of life (Diener et al., 2017). One of the major pursuits of mankind is to find satisfaction and contentment in life, which serves as the key to happiness. Therefore, life satisfaction is desired both as a means to happiness, as well as an end in itself.

It has been argued that life satisfaction can either be determined by a top-down model or a bottom-up model. According to a top-down approach, life satisfaction is pre-determined as a function of genetic make-up and personality traits, while a bottom-up approach implies that a person’s extent of satisfaction with life is situational, one of which is the work domain. Applying the bottom-up approach, life satisfaction gets significantly affected if a person is exposed to work-context stressors such as mistreatment and workplace bullying (Erdogan et al., 2012). Though a number of studies have related workplace bullying with specific aspects of well-being such as stress, depression, anxiety, and health disorders, there is yet a lack of attention on investigating the effect of WB on overall life satisfaction of an individual. Diener et al. (2017) have argued that organizational psychologists need to focus on an overall model of well-being and satisfaction, as well-being at work cannot be separated from other aspects of life and vice versa.

According to the COR theory, people have critical physical and psychological resources that are consumed to deal with demands of life, both work-life and domestic life (Hobfoll and Shirom, 2001). On the other hand, there is always a limited amount of resources at disposal; therefore, resource demand in one domain leads to the availability of lesser resources in other domains (Hobfoll and Freedy, 2017). Resources are described as all those abilities, characteristics, objects, and circumstances that are valued by an individual and are considered to be a threat or potential of threat (Hobfoll, 1989). Drawing from COR theory, this study hypothesizes that WB has an adverse effect on an individual’s psychological resources, one of which is life satisfaction. COR proposes that stressful experiences at the workplace lead to resource depletion, which may lead to adverse behavioral outcomes such as reduced job satisfaction, commitment, and retention. Workplace bullying is one of the most stressful and devastating emotional experience for an individual at the workplace and causes serious mental strain on an individual (Zapf et al., 1996). Building on the same framework, we propose that WB not only depletes psychological resources related to the workplace behaviors but also extends to the other areas of life, predicting overall life satisfaction.

H1: Workplace bullying is negatively related to life satisfaction.

### Mediating Effect of Job Anxiety

Workplace bullying has been clearly related to psychological distress in several studies (Quine, 2003; Tepper, 2007; Einarsen and Nielsen, 2015). In the seminal work on COR theory, it was posited that anything that creates a threat or potential of a threat to valued resources is a cause of stress (Hobfoll, 1989). Workplace bullying causes a threat to all types of valued resources, including psychological resources such as self-esteem, confidence, and mastery, as well as tangible resources, such as employment and resourcefulness. Anxiety, which is defined as “an unpleasant emotional state characterized by concerns, fright, distress, and restlessness that is a response to perceived physical and/or psychological danger” (Fila and Eatough, 2018, p. 155) is experienced in a state of threat to valued resources (De Clercq et al., 2017). Also, anxiety is experienced in situations of high job demands that appear to be beyond control (Hui and Sue-Chan, 2018). WB increases job demands and creates a threat to valued resources and, therefore, significantly contributes to job-related anxiety (De Clercq et al., 2018). Also, it is established that workplace bullying diminished health and well-being (Hoobler et al., 2010; Wagner et al., 2014; Verkuil et al., 2015). In previous works, WB has been individually related to job anxiety and life satisfaction separately. Recently, a study in Pakistan explored the effects of similar oppressive behavior, i.e., despotic leadership and found that it creates anxiety and reduces life satisfaction (Nauman et al., 2018). This study addresses this gap in the literature and examines the mediated relationship of WB, job anxiety, and life satisfaction. Therefore, we propose:

H2a: Workplace bullying is positively related to job anxiety.

H2b: Workplace bullying is related to life satisfaction via job anxiety.

### Mediating Effect of Insomnia

Sleep is a basic necessity of life and has a proven restorative effect (Hobson, 2005). Healthy adults need approximately 7–9 h of sleep to remain optimally functional; those who are not able to maintain these standards of sleep have a high probability of compromised health and well-being (Hirshkowitz et al., 2015).

The word insomnia is derived from the Latin word “*insomnus*” which literally means *no sleep* (Scott and Judge, 2006). Though there are several definitions of insomnia, most constitute three essential elements of sleep quality, sleep latency, and sleep maintenance (Estivill et al., 2003). Sleep disturbance in any of the elements leads to various harmful work outcomes such as negative affect, lack of self-control, unethical behaviors, abuse, and incivility (Barnes et al., 2011, 2016; Barnes, 2012).

Once again reiterating the COR theory, workplace bullying creates significant resource depletion and places disproportionate demands on the psychological and physical resources of the victim. Since sleep is a physiological resource, it gets depleted with exposure to WB. Many targets show signs of post-traumatic stress syndrome, a domain of stress-trauma symptoms that have been found to cause as severe effects as war exposure and imprisonment (Leymann and Gustafsson, 1996; Mikkelsen and Einarsen, 2002). In one study, all the subjects reported depression, insomnia, anxiety, and aggression (Björkqvist et al., 1994). In a recent study, it was examined whether leisure-time physical activity such as running, cycling, or exercise could moderate WB and sleep disturbance relationship, and it was found that it does not (Hansen et al., 2016). This implies that WB is such an intense stressor that physical activity, which otherwise improves well-being and health (Warburton et al., 2006), is unable to take an effect in the presence of WB. This corroborates with other literature which argues that WB acts as a much more powerful stressor than other stressful stimuli (Raja et al., 2017). A close correlate of WB, i.e., abusive supervision leads to sleep deprivation and emotional exhaustion (Han et al., 2017). Moreover, WB reduces the quality of life by causing burnout and work-family conflict (Raja et al., 2017), interpersonal conflict, aggression, and antisocial behaviors (Rhodes et al., 2010). Therefore, employing COR theory, we posit that WB leads to depletion of physiological resource of sleep, causing insomnia, which ultimately leads to depletion of the psychological resource of life satisfaction. Hence, we propose:

H3a: Workplace bullying is positively related to insomnia.

H3b: Workplace bullying is related to life satisfaction via insomnia.

### Sequential Effects of Job-Related Anxiety and Insomnia

In a recent work on COR theory, scholars have discussed its application in the organizational context and chalked out four basic principles of COR theory. The fourth principle termed as Desperation Principle holds that “when people’s resources are outstretched or exhausted, they enter a defensive mode to preserve the self which is often defensive, aggressive, and may become irrational” (Hobfoll et al., 2018, p. 106). Moreover, COR theory also includes an element of momentum, which states that resource loss is not only much more salient than resource gain, it gains momentum and creates exponential resource losses and spill-overs. Using these principles from COR theory, it is argued that WB, which is already conceptualized as a series of oppressive episodes, gains momentum over time (Nielsen and Einarsen, 2012). This aspect of WB creates a compounding effect with spill-overs from loss of one resource to the other. Moreover, a specific application of COR theory to organizational behavior is the job-demand control model (Karasek, 1979), which is further refined as job demand-resource model (Bakker and Demerouti, 2007). Basically, both the models posit that high job demands create resource loss, which leads to job strain, mental and physical ailments and exhaustion. In a similar study, the job demand-control model has been applied to examine the relationship of workplace bullying with depression and insomnia with the mediating effect of job strain (Takaki et al., 2010). Combining COR theory’s desperation and momentum principle and JDC model, we argue that WB creates resource loss, lowers job control, increases job demands, and thereby leads to heightened anxiety (Björkqvist et al., 1994; Lindblom et al., 2006). Anxiety being a stressor itself creates more resource loss leading to poor mental health and insomnia (Rosekind and Gregory, 2010), which in turn gains momentum for further resource loss in the form of reduced life satisfaction (Vázquez et al., 2015; Smyth et al., 2017). In previous literature, these relationships have been tested independently, but to the best of our knowledge, a sequential effect is not yet examined in the literature. Hence, it is posited that WB being a powerful and persistent stressor causes resource loss at multiple levels, because of the element of momentum, thereby triggering a series of resource losses at the physical and psychological level. Therefore, we propose:

H4: Job anxiety and insomnia sequentially mediate the relationship between workplace bullying and life satisfaction.

## Methods

### Sample and Data Collection

We contacted four hospitals located in Lahore, Pakistan, to collect data in order to test our proposed conceptual model. The data were collected without segregating team, department, ward, or shift. The participants were internee junior doctors. The hospital administration allowed our research teams to distribute surveys to their doctor interns in person. We first asked the participants to read a consent form that introduced the study and explained the participant’s rights. Prior to their participation in the surveys, participants signed the consent forms. We informed the participants that their responses would be confidential and their participation would be voluntary without any monetary incentive. Despite the use of convenience sampling technique, which might limit the representation of most of the population, we tried to spread our sample and remain as objective as possible by taking a random sample within the organization *via* our professional contacts. Thus, participants were not nested within the organizations. The study was approved by the Riphah International University Ethical Research Committee.

We collected data at two measurement points in time. In the first wave, we assessed our predictor, workplace bullying, and our first mediator, job-related anxiety, and demographic information. In the second wave after 3 weeks, we measured our second mediator, insomnia, and outcome variable, life satisfaction, from the same respondents, and cross-matched their responses with the first wave. This design helps to curtail potential issues arising from common source bias methods. After filling out surveys each time, participants placed their surveys in sealed envelopes and returned them in person to our research team.

At the first time point, we distributed 320 surveys among doctor interns and received 266 completed surveys, with a response rate of 83%. At the second time point, we targeted those 266 doctor interns and received responses from 211, with a response rate of 79%. Therefore, the average response rate for both these times was 81.2%.

The demographic characteristics we surveyed were gender, education, age, and organization. A summary of the demographic data shows that 48% of the participants were men, *n* = 93, and 58% were women, *n* = 118, and their average age was 24.8 years, SD = 5.98.

### Measures

To measure all scales, we adopted a 5-point Likert response format (ranging from “strongly disagree = 1” to “strongly agree = 5”) instead of the original 6-point Likert scale of insomnia, 7-point Likert scale of life satisfaction, and 4-point Likert scale of job-related anxiety. This modification was based on the researchers’ experience with previous surveys, which indicated that the 5-point format would reduce the frustration level of the respondents, and would thereby increase the response rate and the quality of the responses (Babakus and Mangold, 1992; Sachdev and Verma, 2004; Dawes, 2008).

#### Workplace Bullying

We measured workplace bullying with a 22-item scale developed by Einarsen and Raknes (1997). A sample item is “I have been humiliated or ridiculed in connection with my work.” Participants answered the items on 5-point Likert scale (1 = Never, 5 = daily). The Cronbach’s alpha value for this scale was 0.78.

#### Job-Related Anxiety

We assessed job-related anxiety with a 5-item scale developed by Parker and DeCotiis (1983). A sample item is “I have felt nervous as a result of my job.” Participants answered the items on 5-point Likert scale (1 = strongly disagree, 5 = strongly agree). The Cronbach’s alpha value for this scale was 0.72.

#### Insomnia

To measure insomnia, following Scott and Judge (2006), we employed a 4-item scale developed by Jenkins et al. (1988). A sample item is “I had trouble falling asleep.” Participants answered the items on 5-point Likert scale (1 = strongly disagree, 5 = strongly agree). The Cronbach’s alpha value for this scale was 0.75.

#### Life Satisfaction

To assess life satisfaction, we used the 5-item scale of Pavot and Diener (2009). A sample item is “In most ways, my life is close to my ideal.” Participants answered the items on 5-point Likert scale (1 = strongly disagree, 5 = strongly agree). The Cronbach’s alpha value for this scale was 0.83.

#### Data Analysis

For data analysis, we applied structural equation modeling technique (SEM) and used software (SPSS version 21, and AMOS version 22) to perform data analysis. SEM is one of the best techniques to run more than one analysis at the same time.

#### Control Variables

We applied one-way ANOVA tests to compute the moderation effect of controlled variables like age, gender, and organizations. Results illustrated insignificant variations in life satisfaction, suggesting that the controlled variables had no impact on the interrelationship of the proposed model.

## Results

Table 1 shows means, standard deviations, descriptive statistics, factor loadings and zero-order correlations for all study variables. Correlations between focal variables showed that workplace bullying correlated with the mediators job anxiety, *r* = 0.21, *p* < 0.01, and insomnia, *r* = 0.26, *p* < 0.01. Bullying also correlated with the outcome variable life satisfaction, *r* = −0.26, *p* < 0.01. One of the mediators, job-related anxiety, associated positively with the other mediator, insomnia, *r* = 0.28, *p* < 0.01, and correlated negatively with outcome variable, life satisfaction, *r* = −0.33, *p* < 0.01. Finally, the other mediator, insomnia resulted in a strong negative correlation with life satisfaction, *r* = −0.39, *p* < 0.01.

**Table 1:** Descriptive statistics, factor loadings and zero-order correlations.

Variable	Mean	SD	Factor loading	1	2	3	4
1. Workplace bullying (T1)	3.86	0.41	0.73, 0.86, 0.74	1			
2. Job anxiety (T1)	2.88	0.63	0.57, 0.96, 0.59	0.21^**^	1		
3. Insomnia (T2)	3.26	0.47	0.61, 0.96, 0.58	0.26^**^	0.28^**^	1	
4. Life satisfaction (T2)	2.29	0.67	0.53, 0.68, 0.95, 0.83	−0.26^**^	−0.33^**^	−0.39^**^	1

*N = 211; T1, time 1 T2, time 2*.

** *p < 0.01*.

Table 2 shows the reliability and validity of test findings. For reliability, the omega total and alpha reliability coefficients (Cronbach’s alpha) are reported. Whereas for validity, the convergent validity and discriminant validity were tested.

**Table 2:** Summary of reliability and validity test.

Latent constructs	Omega total	Cronbach’s alpha	CR	AVE	Average correlation with other variables (x)	Discriminant validity (alpha - x)
Workplace Bullying	0.83	0.876	0.82	0.0.61	0.069	0.81
Job anxiety	0.84	0.829	0.76	0.53	0.052	0.78
Insomnia	0.83	0.816	0.77	0.54	0.050	0.77
Life satisfaction	0.88	0.909	0.85	0.60	−0.323	1.23

Omega total and Cronbach’s alpha were used to measure the reliability of scales. Cronbach’s alpha was used to test the internal consistency of the items. All values were greater or equal to cutoff point 0.7 (Pallant, 2010), indicating that all constructs were reliable. On the other hand, omega total assumes that the scale is unidimensional, avoids uncorrelated variances among scales, and estimates the reliability for the composite of items on the scale (which is conceptually similar to Cronbach’s alpha) (McNeish, 2018). Generally, the adequate threshold value for omega total is similar to Cronbach’s alpha at 0.7.

The convergent validity was further tested by the composite reliability (CR) and Average Variance Extracted (AVE). Table 2 shows that all the constructs demonstrated CR and AVE values equal to or greater than their threshold values, respectively (Hair et al., 2010).

Discriminant validity was measured by comparing the Cronbach’s alpha of a construct to its correlations with other model variables (Sila and Ebrahimpour, 2005). According to Ghiselli et al. (1981), if the value of alpha is sufficiently larger than the average of its correlations with other variables, this is evidence of discriminant validity. The difference between the alpha value of each construct and the average correlation of each construct with the other constructs was adequately large: Bullying = 0.81, Job Anxiety = 0.78, Insomnia = 0.77, and Life satisfaction = 1.23. All these values are acceptable for discriminant validity (Sila and Ebrahimpour, 2005).

In order to see the non-dependence of measurement, intraclass correlations ICC1 of four organizations in data were calculated of mediating variables (insomnia and job anxiety) and dependent variable (life satisfaction). ICC1 of mediating variables insomnia and job anxiety and the dependent variable life satisfaction were 0.03, −0.04, and 0.04, respectively, and were below the cutoff point 0.05 (James, 1982). Thus, low ICC1 values, small number of groups (only four organizations), and the lack of intention to conceptualize the research model at group level, individual level analysis was performed to understand the causal relationships.

The SEM was used to test the measurement models and hypotheses of the structural path model. First of all, the measurement model was run for the CFA of all variables. Next, all the direct and indirect paths were checked and their good fit results are shown in Table 3. In our analytical model, we tested a serial mediation.

**Table 3:** Model fit indices for structural path relationships.

Model test	χ2	*Df*	χ2/*df*	CFI	NFI	GFI	TLI	RMR	RMSEA
Direct path from *WB → LS,*	16.304	12	1.359	0.993	0.975	0.979	0.988	0.039	0.041
Direct path from *WB → JA*	8.648	8	1.081	0.998	0.978	0.987	0.997	0.026	0.020
Direct path from *WB → Insm*	12.593	8	1.574	0.988	0.969	0.981	0.978	0.033	0.052
Indirect path from *WB → JA → LS*	44.155	31	1.424	0.984	0.948	0.959	0.976	0.047	0.045
Indirect path from *WB → Insm → LS*	40.684	31	1.312	0.988	0.954	0.964	0.983	0.038	0.039
Indirect path from *WB → JA → Insm → LS*	77.850	58	1.342	0.980	0.928	0.945	0.974	0.045	0.040
Required values			<3	>0.90	>0.90	Close to 1	>0.90	<0.08	<0.06

*WB, workplace bullying; JA, job anxiety; Insm, insomnia; LS, life satisfaction*.

The goodness of fit indices of the proposed model defined as indirect relationship between bullying to life satisfaction *via* job anxiety and Insomnia were χ2/*df* = 1.342, CFI = 0.980, NFI = 0.928, GFI = 0.945, TLI = 0.974, RMR = 0.045, and RMSEA = 0.040. Thus the structural model fits the serial mediation model reasonably by meeting the threshold values as defined by Kline (2015).

**Figure 1:**
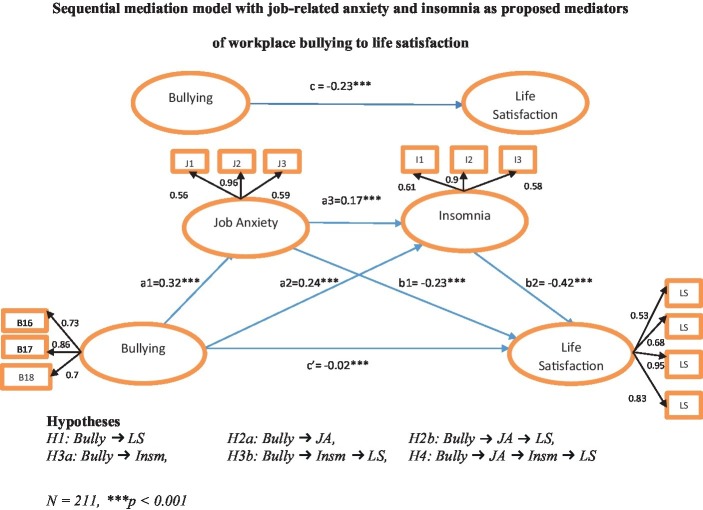
Sequential mediation model.

To test the hypothesis of the proposed model (Figure 1) whether job anxiety and insomnia serially mediated effects of bullying on employee life satisfaction (serial mediation Model 6) by Hayes (2013), we used bootstrap methods. Figure 1 illustrates all possible paths and their related coefficient values. We found that the first path (denoted with c) showed a significant effect on life satisfaction with coefficient value, *b* = −0.23, *p* < 0.001, and supported H1. Moreover, we observed that the direct effect of bullying had a positive and significant impact on job anxiety, *b* = 0.32, *p* < 0.001, and insomnia, *b* = 0.24, *p* < 0.001, respectively, which supported our hypotheses H2a and H3a. Further, the direct effect of job anxiety as first mediating variable, on our second mediating variable, insomnia, *b* = 0.17, *p* < 0.001, was also significant. A review of the direct effects of mediating variables, job anxiety on life satisfaction of the employees, *b* = −0.23, *p* < 0.001, and the effects of insomnia on life satisfaction, *b* = −0.42, *p* < 0.001, were also found significant.

The statistical significance of the indirect effects in the model tested in the current research was examined on 5,000 bootstrap samples. Estimates were taken within a 95% confidence interval, and bias-corrected results are presented in Table 4. It shows that the job-related anxiety mediated significantly between workplace bullying and life satisfaction, *b* = −0.07, SE = 0.04, 95% CI = −0.17 and − 0.02 (H2b supported). Likewise, the indirect effect through insomnia between workplace bullying and life satisfaction was significant, *b* = −0.10, SE = 0.04, 95% CI = −0.20 and − 0.04, which supported H3b. The beta coefficient value was weak; however, prior studies stated the same in which the beta coefficient for indirect effect was 0.08, 0.03 (Liu and Li, 2018), and 0.08 (Qian et al., 2017), and the results were significant as well.

**Table 4:** Tests of direct and indirect effects.

Effect	Coefficient	95% confidence interval (CI)
**Direct effect**		
Workplace bullying → Life satisfaction	−0.23	(−0.43, −0.02)
Workplace bullying → Job anxiety	0.32	(0.11, 0.52)
Workplace bullying → Insomnia	0.24	(0.09, 0.39)
Job anxiety → Insomnia	0.17	(0.07, 0.27)
Job anxiety → Life satisfaction	−0.23	(−0.37, −0.10)
Insomnia → Life satisfaction	−0.42	(−0.60, −0.23)
**Indirect effect**
Workplace bullying → Job anxiety → Life satisfaction	−0.07	(−0.17,−0.02)
Workplace bullying → Job anxiety → Insomnia → Life satisfaction	-0.02	(−0.06, −0.01)
Workplace bullying → Insomnia → Life satisfaction	−0.10	(−0.20, −0.04)
**Total effect**		
Bullying → Life satisfaction	−0.12	(−0.30, −0.11)

*N = 211*.

For testing H4 (denoted with ***c***′), the indirect effect of workplace bullying through job-related anxiety and insomnia was also found to be significant, *b* = −0.02, SE = 0.01, *p* < 0.05, 95% CI = −0.06 to −0.01, having no zero between confidence interval, when both mediating variables were simultaneously entered into the equation. The negative value of *b* revealed that in the presence of both mediators, workplace bullying still has a negative impact on life satisfaction. Thus, results indicated that mediating variables sequentially mediated between workplace bullying and employee life satisfaction.

## Discussion

This study has integrated literature from COR theory and its extensions, i.e., job demand-control model, job demand-resource model, and momentum principle of COR to examine the effect of workplace bullying on life satisfaction directly and indirectly through anxiety and insomnia. All the hypotheses have been supported. Our findings confirm that workplace bullying significantly reduces life satisfaction and the effect is indirectly mediated by anxiety and insomnia.

Previous literature shows evidence of individual relationships of workplace bullying with insomnia (Magee et al., 2015; Hansen et al., 2016; Yuan et al., 2018); WB with anxiety (Leymann and Gustafsson, 1996; Kivimäki et al., 2003; Bowling and Beehr, 2006); anxiety and insomnia with life satisfaction (Rosekind and Gregory, 2010; Smyth et al., 2017; Nauman et al., 2018); and also WB with life satisfaction (Trépanier et al., 2016; Kakarika et al., 2017). However, we have not been able to find, to the best of our efforts, any study on the effect of workplace bullying on overall life satisfaction through the sequential mediating effect of anxiety and insomnia. Therefore, we have demonstrated that workplace bullying is a significant stressor that causes resource losses at multiple levels with spill-overs from one resource to the other.

### Theoretical Contribution

Our study has made a significant theoretical contribution by extending the literature on COR theory, job demand-control, and demand-resources models. Research has focused on the consequences of workplace bullying on individual adverse outcomes related to an individual’s well-being; however, there is a lack of literature on its effect on overall life satisfaction of a person. Diener et al. (2017) have highlighted the lack of research on life satisfaction, and therefore, this study contributes to the literature by addressing this gap and testing the relationship between workplace bullying and life satisfaction.

Our study also extends the literature by showing that job-related anxiety mediates workplace bullying and life satisfaction. It means that workplace bullying triggers a state of threat to resources for the individual, including a threat to employment, career progression, safety, self-esteem, and even to life, which leads to a heightened state of anxiety, which in turn leads to reduced life satisfaction. Furthermore, the study made another contribution by illustrating the mediating effect of insomnia on the relationship between workplace bullying and life satisfaction. This confirmed that episodes of workplace bullying create resource loss. Sleep, an important physiological resource, is depleted in order to conserve the resources lost by workplace bullying, which leads to further resource loss by reducing an individual’s level of life satisfaction.

This study also confirmed the concept of momentum in COR theory (Hobfoll et al., 2018), by demonstrating the effect of workplace bullying on life satisfaction through the sequential mediating effect of anxiety and insomnia. It is demonstrated that workplace bullying leads to anxiety, which in turn leads to insomnia, which again leads to reduced life satisfaction. This confirms the process approach of COR theory (Hobfoll, 1989), showing that stress is not a stand-alone concept. It needs to be observed as a process of affective events that takes place from one level to the other.

Our study showed that the largest effect among all relationships tested was the effect of insomnia on life satisfaction. So every situation, event, or variable that causes insomnia will have an indirect effect over life satisfaction. Workplace bullying alone had a relatively small effect on insomnia. Similarly, job anxiety also had a moderate effect on insomnia and WB and job anxiety also had a similar moderate effect on life satisfaction (for both relationships). Therefore, it showed that only one explanatory variable was not a sufficient predictor of complex phenomena like insomnia and life satisfaction, and a process model is more suitable in explaining the dynamics together.

Finally, this study was conducted among internee doctors employed in house jobs in Pakistani hospitals. This is a unique work setting, as internees are significantly prone to workplace bullying, because of being newly employed, most junior and temporary staff in the hospital. The study validated the hypotheses in a setting of Eastern culture, and in the context of Pakistani organizations. Pakistan is a high-power distance culture (Hofstede, 1983), where it may be assumed that people may be more tolerant toward oppressive behaviors, owing to the phenomenon of learned helplessness (Samnani, 2013). However, it was revealed that high power distance does not nullify the negative effects of WB and it leads to detrimental outcomes both in work and life domain in this cultural setting also.

### Practical Implications for Managers and Policymakers

This study has a number of practical implications. First, the study confirmed the significance of a pervasive and widespread workplace behavior by showing how it affects vital resources in multiple domains and at multiple levels through a momentum and spillover effect. Job anxiety is related to the work domain, whereas insomnia and life satisfaction both belong to the life domain. Therefore, we have shown that a workplace problem does not limit itself in its consequences to the work domain only, but also spreads over to the general life domain of an individual as well. This confirms the matching hypothesis as well as the cross-domain hypothesis (Nohe et al., 2015).

Therefore, it is most important for organizations to identify, prevent, and minimize the occurrence of such behaviors. In high-power distance cultures like Pakistan, it is essential to create a culture of voice (Morrison, 2014), so that victims are able to highlight such an issue as early as possible. Also, organizations should work on the dimensions of organizational virtuousness to foster a culture of social support and positivity (Malik and Naeem, 2016). By focusing on positive behaviors and attitudes, the incidence of such obnoxious episodes can be curtailed. Also, supportive leadership should be present to counter such a problem. Supportive supervisors and leaders reduce job-related stress in leader-member exchange mentoring-protégé relationship (Sosik and Godshalk, 2000), as well lower job-related anxiety in subordinates, thereby improving their work outcomes (Hui and Sue-Chan, 2018).

Second, for policymaking and institutional level frameworks, this study has important implications. In the USA, it was found that 38% to a drastic 90% of employees reported that they experienced WB at some point in their work life (Glendinning, 2001). However, no legislation has been passed to counter this problem. On the other hand, studies in Norway and Finland have reported approximately 8–10% of workers reporting bullying experiences. Yet, the European countries have been very proactive in managing the issue as Norway passed its Work Environment Act in 1994 and Sweden did that in 1997 (Vega and Comer, 2005). In Pakistan, no such legislation exists so far. The Protection against Harassment of Women at Workplace Act was passed in 2010, which particularly provides protection to women against oppressive and harassing environments. However, there is a need to address this issue from a universal perspective. This study showed that WB is a significant workplace behavior, which is experienced by both men and women. Therefore, the government and civil society should realize that there is a need to counter this problem by (1) recognizing it as a potential problem; (2) taking measures for corrective action for perpetrators; and (3) framing protective strategies for potential victims, including mentoring, orientation, and training.

### Limitations and Future Research

Similar to all research inquiries, this study essentially carried certain limitations. One of the major limitations of the study was the use of self-report measures, which creates the issue of common method variance (Podsakoff et al., 2011). However, we attempted to reduce this problem by employing a time-lagged design of data collection. We first collected data for the independent variable, i.e., workplace bullying and one mediator job anxiety. Then, after 3 weeks, we collected responses on the second mediator and dependent variables, i.e., insomnia and life satisfaction. We matched the responses on both time-lagged occasions by collecting information from the same employees at both points in time. By using a time-lagged design, it has been demonstrated that a gap of 3 weeks reduces correlations between predictor and criterion variables by 43% (Johnson et al., 2011).

Another limitation was that data were only collected from internee junior doctors working in public and private sector hospitals in Pakistan. Future research may focus on collecting responses from multiple work settings to make it more generalizable.

Given that the effect of WB on insomnia was small, there is a possibility of other organizational variables such as perceived organizational support, supervisor support, organizational politics, and personal level variables such as emotional intelligence, family support, and work-family conflicts contributing toward insomnia. Future studies may also incorporate more variables such as physical health, income level, and social status to evaluate them as moderating variables.

Future research may also undertake to explore how people with different personality characteristics vary in their coping response to bullying episodes, particularly how personality traits moderate the incidence of anxiety and insomnia. Another possibility for future research is to see how the incidence of workplace bullying varies among men and women, in the Pakistani culture, since it is still a male-dominating culture. Moreover, cross-gender bullying as well as, same-gender bullying may be an interesting topic to explore.

## Conclusion

In conclusion, our study highlights the importance of workplace bullying as a pervasive workplace phenomenon and demonstrates how it affects important outcomes. Our study delineates that workplace bullying is a significant stressor by showing that it increases job anxiety, causes insomnia, and leads to reduced life satisfaction. Our findings confirmed the spillover and momentum effect, as we showed the sequential mediation of job anxiety and insomnia on life satisfaction of an individual caused by workplace bullying among internee junior doctors working in Pakistani hospitals.

## Data Availability Statement

The datasets generated for this study are available on request to the corresponding author.

## Ethics Statement

Research Ethics Committee at Riphah International University has reviewed the aforementioned research proposal and considers the procedures, as described by the applicant, to conform to the University’s ethical standards and RIU guidelines.

## Author Contributions

All authors listed have made a substantial, direct and intellectual contribution to the work, and approved it for publication.

### Conflict of Interest

The authors declare that the research was conducted in the absence of any commercial or financial relationships that could be construed as a potential conflict of interest.
